# The Central Role of Fibrinolytic Response in COVID-19—A Hematologist’s Perspective

**DOI:** 10.3390/ijms22031283

**Published:** 2021-01-28

**Authors:** Hau C. Kwaan, Paul F. Lindholm

**Affiliations:** 1 Division of Hematology/Oncology, Department of Medicine, Feinberg School of Medicine, Northwestern University, Chicago, IL 60611, USA; 2 Department of Pathology, Feinberg School of Medicine, Northwestern University, Chicago, IL 60611, USA; p-lindholm@northwestern.edu

**Keywords:** COVID-19, fibrinolysis, renin-aldosterone-angiotensin-system (RAAS), fibrinolysis, plasminogen activator inhibitor 1 (PAI-1)

## Abstract

The novel coronavirus disease (COVID-19) has many characteristics common to those in two other coronavirus acute respiratory diseases, severe acute respiratory syndrome (SARS) and Middle East respiratory syndrome (MERS). They are all highly contagious and have severe pulmonary complications. Clinically, patients with COVID-19 run a rapidly progressive course of an acute respiratory tract infection with fever, sore throat, cough, headache and fatigue, complicated by severe pneumonia often leading to acute respiratory distress syndrome (ARDS). The infection also involves other organs throughout the body. In all three viral illnesses, the fibrinolytic system plays an active role in each phase of the pathogenesis. During transmission, the renin-aldosterone-angiotensin-system (RAAS) is involved with the spike protein of SARS-CoV-2, attaching to its natural receptor angiotensin-converting enzyme 2 (ACE 2) in host cells. Both tissue plasminogen activator (tPA) and plasminogen activator inhibitor 1 (PAI-1) are closely linked to the RAAS. In lesions in the lung, kidney and other organs, the two plasminogen activators urokinase-type plasminogen activator (uPA) and tissue plasminogen activator (tPA), along with their inhibitor, plasminogen activator 1 (PAI-1), are involved. The altered fibrinolytic balance enables the development of a hypercoagulable state. In this article, evidence for the central role of fibrinolysis is reviewed, and the possible drug targets at multiple sites in the fibrinolytic pathways are discussed.

## 1. Introduction

Infection by the highly contagious coronavirus SARS-CoV-2 has resulted in a global pandemic of coronavirus disease 2019 (COVID-19) [1,2,3]. This virus shares a 79.5% homology to SARS-CoV, the virus responsible for severe acute respiratory syndrome (SARS) [2]. COVID-19 has many clinical and pathologic characteristics in common with SARS and the Middle East respiratory syndrome (MERS) caused by the virus MERS-CoV [4,5,6]. These viruses gain entry into the host cells by attaching a spike protein on their envelope to the angiotensin-converting enzyme 2 (ACE2) expressed on cell surfaces. Clinically, type 2 alveolar cells in the lung are the predominant target, resulting in severe pneumonitis [1,3,6]. In addition, many other organs including the kidneys and cardiovascular system are affected, leading to multiorgan failure. The injury to the lungs is worse in COVID-19 than in SARS or in MERS. In the affected tissues, there is an acute inflammatory response. In the lungs, this manifests as edema, macrophage infiltration and intra-alveolar fibrin deposition, leading to acute respiratory distress syndrome (ARDS) and acute respiratory failure. In autopsied specimens, diffuse alveolar damage, microthrombi in perialveolar vessels and intra-alveolar hemorrhage have been observed [7,8,9,10,11,12,13]. The clinical course is associated with a hypercoagulable state, resulting in thrombotic complications in arteries, veins, catheters, arteriovenous fistulas and implantable devices such as the ECMO (extracorporeal membrane oxygenation circuit). In all these aspects, the fibrinolytic system is involved. The fibrinolytic system is activated by acute tissue injury and inflammation and is an important element in the body’s response to these acute viral infections. In this article, the evidence of the participation of various components of the fibrinolytic system in the pathogenesis of these disorders is reviewed. In addition, interference with specific sites of the fibrinolytic pathways may mitigate the tissue injury and thus they are compelling drug targets.

## 2. The Fibrinolytic System (Aka Plasminogen-Plasmin System)

**Figure 1:**
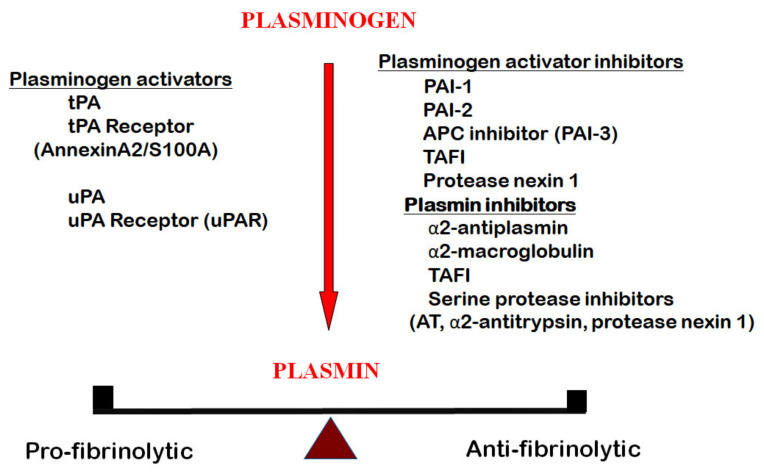
The fibrinolytic system, aka the plasminogen-plasmin system, consisting of the serine protease plasmin, derived from plasminogen by the activation of its activators, tissue plasminogen activator (tPA) and urokinase-type plasminogen activator (uPA). These two activators are ligands to their cellular receptors, urokinase receptor (uPAR) and a heterotetramer annexin, A2/S100A, respectively. The plasminogen activators and plasmin are inhibited by plasminogen activator inhibitors, plasminogen activator inhibitor 1 (PAI-1), plasminogen activator inhibitor 2 (PAI-2), activated protein C inhibitor (APC) inhibitor (PAI-3), thrombin activatible fibrinolysis inhibitor (TAFI) and protease nexin 1. Plasmin is inhibited by α2-antiplasmin, α2-macroglobulin, TAFI and several serine protease inhibitors (AT, α2-antitrypsin, protease nexin 1).

The chief component of this system is a serine protease, plasmin. It is formed by the activation of its precursor plasminogen (Figure 1) [14,15]. In humans, the two activators are tissue type plasminogen activator (tPA) and urokinase-type plasminogen activator (uPA). They bind to their respective receptors on cell surfaces. The receptor for tPA is a heterotetramer complex of annexin A2 and a surface binding protein S100A10 [16,17], whereas that for uPA is urokinase receptor (uPAR) [18]. To maintain a physiologic balance, the fibrinolytic system is regulated by serine protease inhibitors (serpins) at various activation sites. Those inhibiting the conversion of plasminogen to plasmin are plasminogen activator inhibitor 1 (PAI-1), plasminogen activator inhibitor 2 (PAI-2), activated protein C inhibitor (APC) inhibitor (PAI-3), protease nexin 1 and defensin (for tPA only). Those that inhibit plasmin are α2-antiplasmin, α2-macroglobulin, as well as several serine proteases, including antithrombin, α2-antitrypsin and protease nexin 1. In addition, thrombin activatable fibrinolytic inhibitor (TAFI), a carboxypeptidase, prevents plasminogen binding and plasmin formation by cleaving lysine residues on fibrin. Among the inhibitors of plasmin, α-antiplasmin [19] and PAI-1 [20] are the most effective ones. The components of the fibrinolytic system regulate many physiologic functions and participate in the pathogenesis of numerous pathologic disorders. tPA regulates fibrin formation in blood and takes part in many functions in the brain. On the other hand, uPA and its receptor uPAR are involved in inflammation, tissue repair, cell proliferation and a multitude of other body functions. In particular, the uPA and uPA/uPAR complex is highly expressed in the airway epithelium in the lung [21,22] and plays an important role in acute lung injury. Under physiologic conditions, the activators and inhibitors are in a state of balance and regulate hemostasis. This balance is deranged in COVID-19, as discussed below.

## 3. Invasion of SARS-CoV-2 into Host Cells and Subsequent Events

**Figure 2:**
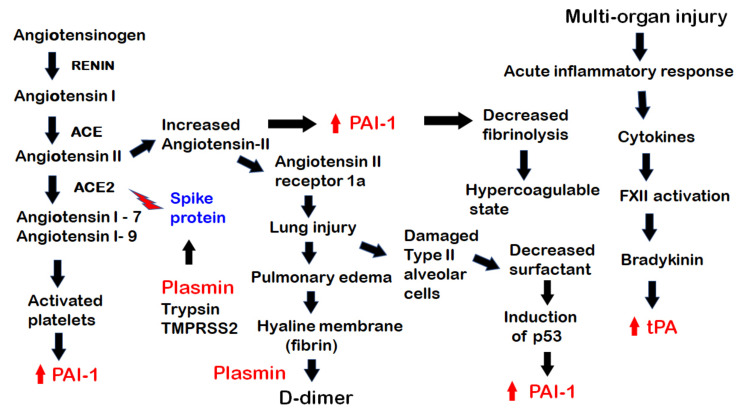
The pathogenesis of COVID-19, showing the involvement of components of the fibrinolytic system in various steps. From the left, the renin-aldosterone-angiotensin-system (RAAS) pathway is shown. Plasmin and other proteases, trypsin and TMPRSS2 act on the SARS-CoV-2 spike protein to facilitate its binding to ACE2 on the surface of host cells. With the binding, the virus invades the host cells while ACE2 is internalized and unable to process the breakdown of angiotensin II, leading to its excess. The excess of angiotensin II leads to an increase in PAI-1 and decreased fibrinolysis, creating a hypercoagulable state, while the excess of angiotensin II binds to its receptor angiotensin II receptor 1a, causing lung injury and leading to pulmonary edema with the formation of a hyaline membrane with fibrin in the alveoli. This is broken down by plasmin with the formation of D-dimer. The diffuse alveolar damage with damaged type II alveolar cells leads to decreased surfactant, which results in induction of the p53 pathway and increased in PAI-1. Of note (on the left), platelets are activated by angiotensin 1–9 and by other pathways. They then release PAI-1 into the circulation. There is also an acute inflammatory response with multi-organ injury. Inflammatory cytokines activate factor XII leading to bradykinin formation and a subsequent increase in tPA.

SARS-CoV-2 is highly contagious and is transmitted both as droplets and as aerosols. The virus gains entry to the host cells by attaching a spike protein on the viral envelop to angiotensin converting enzyme 2 (ACE2) on the cell surface. ACE2 is an integral component of the renin-aldosterone-angiotensin-system (RAAS) (Figure 2) [23,24]. In SARS-CoV-2 infection, the pathogenesis involves the following steps. First, the RAAS is an essential regulator of vascular functions including blood pressure, sodium balance and blood volume. In the RAAS, a plasma protein, angiotensinogen, is converted by a renal aspartic protease renin to angiotensin I. Angiotensin I is then metabolized by angiotensin converting enzyme (ACE) to angiotensin II. Angiotensin II is further metabolized by ACE2, a homolog of ACE [25], producing a vasodilator, angiotensin 1–7. ACE2 is expressed on cell membranes in the lung in the trachea and bronchial epithelial cells, type 2 alveolar cells and macrophages, in the kidney on the luminal surface of tubular epithelial cells, in the heart endothelium and myocytes, in the gastrointestinal epithelial cells, in the testis and in the brain [26,27,28,29,30]. ACE-2 acts as the receptor for SARS-CoV-2, as well as for other coronaviruses such as SARS-CoV [31,32], by binding to the spike protein on the viral envelop. This binding requires the proteolysis of the S1/S2 cleavage site of the spike protein by proteases in the pulmonary airway, including plasmin, furin, trypsin and transmembrane proteases (TMPRSS2) [33,34], for entry into host cells. Following its attachment to the spike protein, ACE2 is internalized and downregulated [35,36]. The reduction in ACE2 results in a diminished degradation of angiotensin II, leading to a buildup of angiotensin II. This produces several deleterious effects. Angiotensin II binds to a cellular receptor angiotensin II type 1a receptor in the lung, causing acute lung injury [26,37,38]. This injury is characterized by pulmonary edema, infiltration of monocytes and macrophages, diffuse alveolar damage, along with increased fibrin deposition, hyaline membrane formation and microvascular thrombosis [7,13,39,40]. Autopsy specimens of lungs from COVID-19 patients showed a high prevalence of diffuse alveolar damage, capillary congestion and capillary microthrombi [41]. There is severe capillary endothelial injury with widespread capillary fibrinous microthrombi [42]. The lungs of COVID-19 patients contain a much higher load of capillary microthrombi and less thrombi in post-capillary venules than those in influenza patients. Pulmonary capillary microthrombi are also found in association with complement components [43] and neutrophil extracellular traps (NETs) [44]. Proteolytic breakdown of the fibrin results in the generation of D-dimer [45,46]. The magnitude of D-dimer has been correlated to the severity of the infection [45]. Angiotensin II also increases the expression of PAI-1 in endothelial cells [47,48,49], resulting in decreased fibrinolysis and contributing to a hypercoagulable state. The lung injury occurs with the viral entry into the type II alveolar cells. As these cells are the source of surfactant, there is loss of surfactant. The decrease in surfactant leads to the induction of the p53 pathway. p53 binds to uPA/uPAR mRNAs and suppresses their expression while increasing PAI-1mRNA [50,51]. The diminished effect of ACE2 also leads to decreased formation of angiotensin 1–7 and angiotensin 1–9. Angiotensin 1–7 has been shown to impair the release of PAI-1 by cultured endothelial cells in vitro [52]; hence, a diminished angiotensin 1–7 will result in more PAI-1. On the other hand, angiotensin 1–9 increased PAI-1 and thus the thrombotic tendency [53]. In addition, angiotensin 1–9 activates platelets with the release of PAI-1 from their α-granules. The sum effect is enhanced PAI-1 activity. PAI-1 contributes to many changes in the lung. It keeps in check excessive fibrinolysis and lessens the risk of progression of the alveolar damage to intra-alveolar hemorrhage. PAI-1 further enhances epithelial–mesenchymal transition and fibrosis [50,51,54,55,56]. The infiltration of monocytes and macrophages evokes an acute inflammatory response, with elevated levels of proinflammatory cytokines such as interleukin (IL)-6, IL-1, tumor necrosis factor (TNF)α and IL-8. In many of the severe cases, an uncontrolled and continuous interaction between natural killer (NK) cells of the innate immune system and CD8 positive cytolytic T cells of the adaptive immune system leads to a cytokine storm with very high levels of serum pro-inflammatory cytokines and ferritin [57,58]. The inflammatory cytokines activate the intrinsic pathway of the coagulation cascade, with factor XII activation and progression to thrombin generation. A thrombo-inflammatory condition then ensues. These inflammatory cytokines significantly upregulate the expression of PAI-1 [59].

The role of PAI-1 has been extensively studied in many pathological conditions. In health, PAI-1 originates from endothelial cells, platelets, liver, adipose tissues and macrophages [60]. Though PAI-1 is present in an active conformation, especially in platelets, it can be readily converted to the inactive latent form. PAI-1 is well recognized to play an important role in the pathogenesis of a wide variety of conditions, including aging, cellular senescence, obesity, cardiovascular disease, hypertension, diabetes, fibrosis and thrombosis [20,56,61,62,63,64,65], It is notable that people with many of these conditions are more susceptible to COVID-19 and have worse outcomes [66].

## 4. Hypercoagulability

A marked increase in thrombotic complications due to hypercoagulability has been observed in patients with COVID-19. Thrombosis had been observed in both superficial and deep veins, in arteries and in the microvasculature. One remarkable example is acute ischemic strokes in young patients with no previous arterial disease [67]. The acute lung injury in COVID-19 patients shows fibrin deposits in the pulmonary microcirculation, forming microthrombi, which are thought to be due to viral injury to the endothelium [68]. Thrombosis is more common in the severely ill patients in the ICU, with a notably greater incidence of pulmonary embolism [69,70,71].

Microthrombi have been observed clinically in multiple organs: in the heart, resulting in acute myocardial infarction [72], within tumors [73], in the lungs [74,75] and in the kidneys, resulting in acute renal failure [76,77], whereas autopsy findings have revealed more widespread microthrombosis elsewhere [9,12,13,39,77,78]. Heart failure has been found in almost a third of hospitalized COVID patients and is the second leading cause of death following ARDS [79]. Notably, the microthrombi were found to be rich in platelets and megakaryocytes [80].

The coagulation profile in blood shows mild prolongations in prothrombin time (PT) and activated partial thromboplastin time (APTT), as well as mild thrombocytopenia, and does not reflect the in vivo state of hypercoagulability. On the other hand, a high fibrinogen and a high D-dimer are characteristic of patients with COVID-19 [45,81,82,83,84]. The acute inflammatory response in the infection accounts for some of the increase in the fibrinogen levels. In response to acute inflammation, the hepatic synthesis of fibrinogen has been shown to increase two- to ten-fold as an acute phase reactant [85]. The increase in fibrinogen was found to be proportional to the severity of the disease [45,86]. The D-dimer increase is observed in most patients and found to be correlated not only with the severity of the infection but also with thrombotic events [45,86]. Since D-dimer is the product of plasmin degradation of cross-linked fibrin [46] and since fibrinolytic activity is low [87,88], the enigma of the high D-dimer has not been fully elucidated [89]. Based on observations in acute lung injury and acute respiratory distress syndrome in SARS-CoV, it is generally believed that the major portion of the circulating D-dimer originates from the pulmonary lesion [90,91]. Continuous fibrin deposition into the alveoli with its breakdown by plasmin leads to the production of D-dimer. D-dimer has been recovered in the bronchoalveolar lavage (BAL) fluid of patients with acute respiratory distress syndrome (ARDS), indicating that intra-alveolar coagulation and fibrinolysis occur in this syndrome [92]. However, in this study, plasma and BAL D-dimer levels did not correlate. The generation of D-dimer occurs despite the antifibrinolytic effects of thrombin activatable fibrinolysis inhibitor (TAFI), protein C inhibitor (aka PAI-3) [93] and PAI-1 [40,94]. The APTT is often prolonged with the presence of lupus anticoagulant [95,96]. Factor XII is frequently low, partly due to loss in the exudation in pulmonary lesions, as a high level of this factor was found in BAL [97]. Activated factor XII has high homology to tPA and can activate plasminogen to plasmin [98]. Thus, a low factor XII would contribute to reduced fibrinolysis in this setting.

Although each of the individual conventional clotting tests does not give a whole picture of hemostasis, a more complete hemostatic picture can be found using whole blood testing in viscoelastography (VE). Two common techniques are thromboelastography (TEG) and rotational thromboelastometry (ROTEM). The progressive increase in viscosity of clotting whole blood and subsequent clot lysis is plotted graphically. In TEG, the time taken for a clot to form is recorded as the r value, and the velocity of clot formation as K (minutes) and K (angle). The clot strength is the maximum amplitude (MA), whereas clot lysis at 30 min is Lys30. Short r and K values, with increased MA, were found in COVID-19 patients, whereas in most of the ICU patients no clot lysis was seen at 30 min [87,88]. These findings indicate a hypercoagulable state. They were correlated with a high D-dimer and severity of the disease. The absence of fibrinolysis characterizes a pathologic hypercoagulable condition often associated with thrombosis, showing decreased viscoelastic fibrinolysis associated with elevated D-dimer and plasmin-antiplasmin (PAP) complexes [87]. Those patients with fibrinolysis shutdown had a 40% incidence of VTE compared to 5% in those without shutdown [87]. Fibrinolysis shutdown with a high D-dimer level is also correlated with renal failure [87]. Similar findings were seen using the ROTEM [99,100,101,102]. In a systemic review of ten studies of 389 COVID-19 patients, 90% of which were severe, the above-described hypercoagulability and fibrinolysis shutdown were present [103]. These patients were also on anticoagulant therapy. However, the mechanism by which these changes occur are not clearly shown. Obviously, many more studies are needed to provide a clear-cut explanation. Results of these studies would enable a better use of viscoelastography in guiding the use of anticoagulation therapy.

Both TEG and ROTEM have the advantage of being readily available at the point-of-care, with a short turnaround time for the results. It can also be used to guide anticoagulant therapy.

The fibrinolysis shutdown had previously been shown to be a poor prognostic indicator in acute sepsis and in severe trauma [104].

## 5. Role of Platelets

Recent observations in COVID-19 patients revealed that platelet activation takes place. Platelets form aggregates with neutrophils, monocytes and T-lymphocytes. Monocyte aggregates were found to release tissue factor [104,105], which can activate platelets. The activation of platelets is correlated to the severity and poor outcome of the disease [105]. The evidence for platelet activation were alterations in the gene transcriptome; increased P-selectin; and increased platelet aggregation to ADP, thrombin and collagen [106]. In addition, there was increased adhesion and spreading of platelets on fibrinogen and collagen. These changes contribute to the hypercoagulability not only through increased platelet activation but also through increased tissue factor release. In addition, platelets are the richest source of PAI-1 in the circulation. Activated platelets release PAI-1 from the α granules, as has been shown in trauma patients [107]. However, it is not known if PAI-1 is similarly released by platelets in COVID-19 infection.

## 6. Activation of the Complement System

One major innate immune response is the complement system [108,109]. It is composed of protein elements that can be activated by three different pathways—classic, alternative and lectin. Each pathway is activated respectively by the antigen/antibody complex, spontaneous hydrolysis of C3 and the mannose-binding lectin (MBL)–mannose complex. Following activation, C3 convertase cleaves C3 to C3a and C3b, resulting in the generation of C5 convertase, which cleaves C5 to C5a and C5b. C5b then forms a complex with other complement proteins to generate the membrane attack complex (MAC), consisting of C5b-C6-C7-C8-C9 (often abbreviated as C5b–9). MAC acts on cells by disrupting the cell surface, resulting in cell lysis. There are complement control proteins in the plasma that regulate the complement system. They are C1-inhibitor (C1-INH) which binds to C1 and prevents its activation, decay accelerating factor (CD55), membrane cofactor protein (CD46), protectin (CD59), complement C3b/C4b receptor 1, CR1 (CD35) and factor H.

The complement system has been shown to be activated in animal models of SARS-CoV and MERS-CoV infections [40,110,111] and was observed in COVID-19 patients [43]. Autopsy examination of COVID-19 patients using immune-histochemical staining revealed the presence of mannose-binding lectin-associated serine protease (MASP-2), C4d (lectin pathway) and C5b–9 (membrane attack complex) in the microvasculature of the lung in the alveolar septa and in the skin. These complement components colocalize with SARS-CoV-2. In both biopsy and autopsy materials, vascular lesions with microthrombi were found, along with C4d and C5b–9.

These observations are highly significant since the complement system is linked to both the coagulation and the fibrinolytic systems. They support another explanation for the high incidence of thrombotic complications in COVID-19.

**Figure 3:**
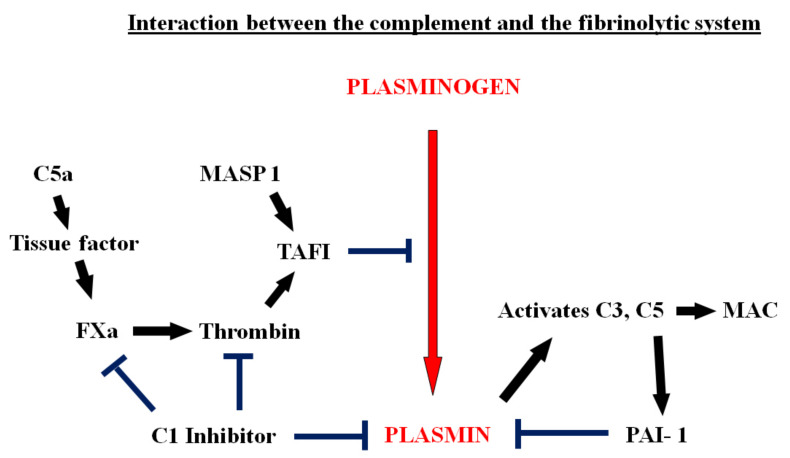
The interactions of the complement system with coagulation and fibrinolysis. C5a activates tissue factor, initiating the coagulation cascade, leading to thrombin generation and the formation of thrombin-activatable fibrinolysis inhibitor (TAFI). TAFI blocks the conversion of plasminogen to plasmin. TAFI can also be activated by another component of the complement system, MASP-1. C1 inhibitor blocks the activation of coagulation and inhibits plasmin. In addition, plasmin activates C3 and C5 directly and this leads to formation of the membrane attack (MAC). C5a also increases the expression of PAI-1 in mast cells.

There is crosstalk between members of the complement system and coagulation factors on the one hand, and components of the fibrinolytic system on the other [109], and this is illustrated in Figure 3. C5a is a procoagulant and activates tissue factor, and suppression of C3 or of C5 reduces tissue factor activity. With activation of tissue factor, the extrinsic pathway of the coagulation cascade is triggered, leading to thrombin generation. Thrombin converts a carboxy-peptidase B-like proenzyme to thrombin-activatable fibrinolysis inhibitor (TAFI), which blocks the conversion of plasminogen to plasmin [112]. TAFI can also be activated by another component of the complement system, MASP-1 [113,114]. The inhibitor of complement activation, C1-protease inhibitor(C1-inh), blocks the activation of coagulation and of complement [115]. It also inhibits plasmin [116]. In this complex setting, activation of the complement system affects fibrinolysis and vice versa. Plasmin has been shown to activate C3 and C5 directly and lead to formation of the membrane attack (MAC) [117]. C5a has been shown to increase the expression of PAI-1 in mast cells [118].

In addition, C3a and C5b–9 were found to activate platelets [119,120,121]. Whether or not PAI-1 is released is not known. If it does, this will be an additional pathway by which complement activation may inhibit fibrinolysis.

Complement components have been found to colocalize in microvasculature with SARS-CoV-2, activate coagulation and inhibit fibrinolysis, thus raising the question of whether eculizumab or related C5 cleavage targeted therapies would be useful to prevent detrimental thrombosis and microvascular injury [43]. However, in a case report of a patient with preexisting atypical hemolytic syndrome (aHUS) who contracted severe COVID-19, eculizumab therapy did not prevent severe endothelial injury or D-dimer elevations [122]. Nonetheless, treatment that inhibits the complement system remains an attractive drug target. Two approved drugs are eculizumab and the longer acting ravulizumab, both being monoclonal antibodies with high affinity to C5 and preventing the cleavage of C5 to C5a, thus blocking the formation of C5–9. In addition, there are other agents blocking C3 activation under development. One of these is narsoplimab, a monoclonal antibody against MASP-2 [123].

## 7. Fibrinolytic Balance

**Figure 4:**
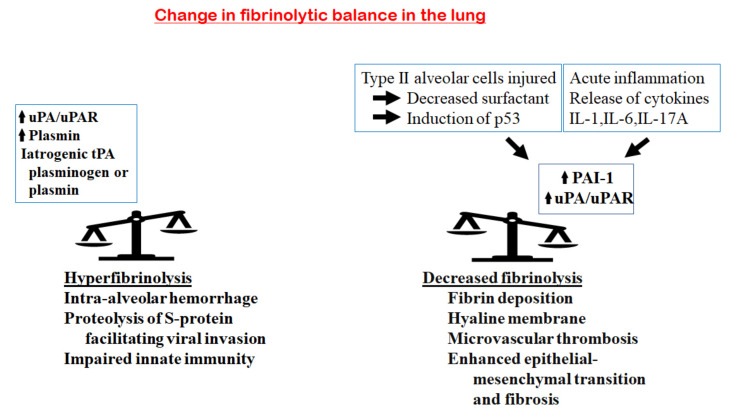
Changes in the fibrinolytic balance in the lung in COVID-19. On the one hand, an increase in fibrinolytic components, including uPA/UPAR, plasmin, iatrogenic tPA, plasminogen or plasmin, would tilt the balance towards hyperfibrinolysis. This enhances the proteolysis of the viral S-protein and facilitates viral invasion; increases the breakdown of fibrin in the alveoli, generating more D-dimer; enhances the risk of intra-alveolar hemorrhage and impairs the innate immunity of host cells. On the other hand, the acute inflammatory response in the lung releases inflammatory cytokines (IL-1, IL-6 and IL-17A), leading to an increase in PAI-1 and a decrease in the uPA/uPAR complexes. In addition, injury of the type II alveolar cells results in decreased surfactant and the induction of the p53 pathway that upregulates PAI-1. These will tilt the balance to hypofibrinolysis, with the consequences of increased fibrin deposition, hyaline membrane formation, microvascular thrombosis, enhanced epithelial–mesenchymal transition and fibrosis.

Under physiological conditions, the body’s fibrinolytic components are kept in a state of balance between the profibrinolytic and the antifibrinolytic factors (Figure 1). This delicate balance can be perturbed under many pathological conditions. In COVID-19, an abnormal fibrinolytic balance is one of the major players in its pathogenesis. As the lung is the most commonly involved organ, we have examined the local changes in the fibrinolytic components and their effects on the fibrinolytic balance (Figure 4).

Studies in the epithelial cells in the normal lung have indicated that uPA, its receptor uPAR and PAI-1 are expressed [22,124,125]. This state of fibrinolytic balance keeps the airways and alveoli free from fibrin deposition. In healthy subjects, uPA and uPAR are present in the bronchoalveolar lavage fluids with no procoagulants, keeping the airways and alveoli clear. tPA is not involved. There is a temporal relationship between COVID-19 infection and fibrinolysis. In the acute phase of the infection, the inflow of inflammatory fluids containing fibrinogen and coagulation factors leads to fibrin deposition and hyaline membrane formation. The acute inflammatory cytokines consisting of IL-1, IL-6 and IL-17A upregulates PAI-1 and suppresses the expression of uPA and uPAR [50,51].

The relationship between lung injury and changes in uPA, uPAR and PAI-1 was fully demonstrated in a bleomycin-induced model in mice [50]. Following injury to type II alveolar cells by bleomycin, there is suppression of uPA and uPAR, along with an increase in PAI-1. As uPA induces alveolar epithelial cell proliferation, these changes promote apoptosis and fibrosis. The bleomycin lung injury also induces p53 expression, leading to further downregulation of uPA and uPAR and upregulation of PAI-1 [125]. Similar changes were observed in lungs injured by smoking [50].

In COVID-19 infection, there is injury to the type II alveolar cells, with the same results of increased p53 expression, suppression of uPA, uPAR and an increase in PAI-1. In addition, the type II alveolar cells are the source of surfactant. The damage by the viral infection leads to a marked reduction in surfactant. With the decreased surfactant, the p53 pathway is induced, leading to an increase in PAI-1 and a decrease in uPA and uPAR [51,126]. The fibrinolytic balance is then shifted to a hypofibrinolytic state. This enhances fibrin deposition, hyaline membrane formation and microvascular thrombosis. In the later phase of the infection, the lung epithelial cells undergo epithelial–mesenchymal transition [127] and fibrosis [128]. These changes are also enhanced by PAI-1 [54].

## 8. Bleeding Complications

In contrast to thrombosis, bleeding in COVID-19 patients has received less attention in the current literature. The incidence ranges from 6–21% in hospitalized patients [129,130,131,132]. The largest retrospective study, with 102 hospitalized patients anticoagulated on therapeutic doses, showed an incidence of 11%, well correlated to mortality. There was little or no increase in fibrinolysis. The etiology of the bleeding is likely endothelial damage from the viral infection. The main risk factors for bleeding are the use of antiplatelet drugs, anticoagulation and underlying vascular lesions. A recent report of major bleeding in anticoagulated patients with macrothrombosis prompted caution in using therapeutic doses of heparin and recommended close monitoring [133].

## 9. Therapeutic Targets

As impaired fibrinolysis due to increased PAI-1 levels was believed to be involved in the pathogenesis of acute lung injury [22], various fibrinolytic agents have been used in its management. These agents include tPA, uPA, plasminogen and plasmin. A meta-analysis of 22 studies of fibrinolytic therapy in animal models of acute lung injury revealed that the lung injury, oxygenation, local neutrophil infiltration and mortality are improved [134]. A similar approach is being employed to mitigate the clinical course of COVID-19 using various components of the fibrinolytic system. Inhalation of plasminogen was found to improve the lung lesions and hypoxemia in 13 patients with COVID 19 [135]. To augment fibrinolysis, tPA has been used and delivered intravenously in ongoing clinical trials [136,137]. Another strategy employs small molecules that inhibit PAI-1, one of which is TM5411 [62,138]. At the time of this writing, it is also is undergoing clinical trial. Results of these trials will verify the adverse role of PAI-1 in COVID-19.

## 10. Conclusions

This review of the pathogenesis of COVID-19 provides findings in the published literature showing that the components of the fibrinolytic system are involved in multiple steps of the viral infection. This involvement ranges from the invasion of the host by the virus to organ damage and a variety of complications, including thrombosis and fibrosis. Plasmin processes the viral S-protein for its entry into the host cells. The subsequent binding of the S-protein to ACE-2 triggers a rise in angiotensin II, which upregulates PAI-1. The lung injury with edema, hyaline membrane formation and alveolar damage are examples of the ways in which fibrinolysis is heavily involved. Other changes such as monocyte and macrophage infiltration in the lesions, evoking an acute inflammatory and cytokine response, further enhance the fibrinolytic changes, especially an increase in PAI-1. This close relationship between fibrinolysis and the disease process offers an opportunity for a therapeutic target.

The pathogenesis of this viral infection also involves the activation of the complement system. Notably, there is interaction among coagulation activation, with tissue factor initiating the coagulation cascade and thrombin generation, TAFI activation and the inhibition of fibrinolysis. Fibrinolysis is also inhibited by TAFI. Several steps in the complement activation pathways also inhibit fibrinolysis. These actions form an attractive basis to explain the hypercoagulability in COVID-19, with its increased incidence of thrombotic complications. It is also notable that the degree of fibrinolytic involvement varies with the severity of the illness and furthermore may change temporally during the course of the disease. As such, the therapeutic approach should be appropriately tailored to the specific phases of the clinical course. We expect that ongoing clinical trials will verify the role of altered fibrinolysis in the pathogenesis of COVID-19.
